# Health-Related Quality of Life in Patients Living with Wilson Disease in Spain: A Cross-Sectional Observational Study

**DOI:** 10.3390/jcm12144823

**Published:** 2023-07-21

**Authors:** Zoe Mariño, Marina Berenguer, Luis Peña-Quintana, Antonio Olveira, Anna Miralpeix, Isabel Sastre, Ana Reyes-Domínguez, Pilar Castillo, Clàudia García-Solà, Ariadna Bono, Miriam Romero, Francisco Javier Pérez-Sádaba, Susana Aceituno, Anna Anguera

**Affiliations:** 1 Liver Unit, Hospital Clínic Barcelona, Instituto de Investigaciones Biomédicas August Pi i Sunyer (IDIBAPS), Centro de Investigación Biomédica en Red de Enfermedades Hepáticas y Digestivas (CIBERehd), European Reference Networks (ERN)-RARE Liver, University of Barcelona, 08007 Barcelona, Spain; amiralpeix@clinic.cat (A.M.); clgarcias@clinic.cat (C.G.-S.); 2 Department of Gastroenterology and Hepatology, Hospital Universitari i Politècnic La Fe, Centro de Investigación Biomédica en Red de Enfermedades Hepáticas y Digestivas (CIBERehd), Instituto de Investigación Sanitaria (IIS La Fe), 46026 Valencia, Spain; marina.berenguer@uv.es (M.B.); isa.sastre99@gmail.com (I.S.); ariadna_bono@iislafe.es (A.B.); 3 Pediatric Gastroenterology, Hepatoloy and Nutrition Unit, Complejo Hospitalario Universitario Insular Materno-Infantil, 35016 Las Palmas de Gran Canaria, Spain; luis.pena@ulpgc.es (L.P.-Q.); anitard71@gmail.com (A.R.-D.); 4 Centro de Investigación Biomédica en Red de la Fisiopatología de la Obesidad y Nutrición (CIBEROBN), Instituto de Salud Carlos III, University of Las Palmas de Gran Canaria, 35001 Las Palmas de Gran Canaria, Spain; 5 Department of Gastroenterology, Hospital La Paz, 28046 Madrid, Spain; aolveiram@gmail.com (A.O.); pcastillograu@gmail.com (P.C.); miriam.romero.portales@gmail.com (M.R.); 6 Outcomes’10 SLU, 12071 Castellón de la Plana, Spain; fperez@outcomes10.com (F.J.P.-S.); saceituno@outcomes10.com (S.A.); 7 Medical Department, Alexion, AstraZeneca Rare Diseases, 08028 Barcelona, Spain; anna.anguera@alexion.com

**Keywords:** Wilson disease, health-related quality of life, real-world evidence, emotional status, adherence

## Abstract

Wilson disease (WD) is a rare copper metabolism disorder caused by mutations in the *ATP7B* gene. It usually affects young individuals and can produce hepatic and/or neurological involvement, potentially affecting health-related quality of life (HRQoL). We assessed HRQoL in a cohort of Spanish patients with WD and evaluated disease impact on several domains of patients’ lives, treatment adherence, drug preference and satisfaction, and healthcare resource utilisation in a cross-sectional, retrospective, multicentric, observational study. A total of 102 patients were included: 81.4% presented isolated liver involvement (group H) and 18.6% presented neurological or mixed involvement (group EH). Up to 30% of patients reported a deteriorated emotional status with anxiety and depression, which was greater in the EH subgroup; the use of neuropsychiatric drugs was high. Over 70% of the patients were satisfied with their current treatment but complained about taking too many pills, stating they would consider switching to another more patient-friendly treatment if available. The Simplified Medication Adherence Questionnaire revealed only 22.5% of patients were fully adherent to therapy, suggesting that alternative therapies are needed. This real-world study, even though is highly enriched with hepatic patients and mild disease, shows that WD impacts patients’ HRQoL, especially in the emotional domain.

## 1. Introduction

Wilson disease (WD) is a rare autosomal recessive disorder of copper metabolism [1]. Most patients with WD are diagnosed during childhood and youth [2,3], with disease prevalence reported at 1:30,000–50,000 cases worldwide [4,5].

WD is caused by a mutation in the *ATP7B* gene, which encodes a transmembrane copper-transporting ATPase responsible for copper incorporation into ceruloplasmin and excess copper excretion from hepatocytes into the bile [6]. Reduced ATP7B protein levels resulting from mutations in this gene lead to copper retention and deposition in several organs, including the liver, brain, kidneys, and corneas [5,7]. WD is thus a highly heterogeneous disease with several phenotypes [3], which include hepatic, neurological, and psychiatric involvement or a mixed combination of these manifestations [8]. Hepatic manifestations are the most frequent (up to 70%). Liver involvement is the norm among children and can vary from asymptomatic, presenting only biochemical abnormalities, to acute hepatitis or liver failure, chronic hepatitis, or clinical decompensation of cirrhosis [5,6]. Neurological and psychiatric symptoms are more frequent in young adults aged 20–30 years [9], affecting approximately 20–65% of patients with WD [10]. The most frequent neurological symptoms are tremor, dystonia, Parkinsonism, dysarthria, dysphagia, drooling, and epilepsy [11]. Psychiatric symptoms can arise before, concurrent with, or after WD diagnosis and treatment [12], and may include personality changes, mood (mania, depression, anxiety) and sleep disturbances, cognitive deficits, and psychosis [9,11]. It is estimated that up to 70% of patients with WD may present with psychiatric involvement at some time [13].

In order to improve the patient’s disease prognosis and decrease potential complications, early diagnosis and treatment are crucial; timely therapy is associated with reduced morbimortality and prevention of cirrhosis or liver transplantation [14]. The overall goal of therapy for patients diagnosed with WD is to reduce copper levels and prevent its accumulation in the aforementioned organs by balancing copper intake and excretion [6,14] with conventional drug therapy removing excess copper.

With the appropriate treatment, most patients present long-term survival with life expectancy similar to the general population [15]; however, current therapies can be associated with adverse events, including paradoxical neurological deterioration [5,6]. In addition to safety issues, current therapies have problematic posology, hampering compliance over time. In this light, safer therapies and more suitable regimens are needed [6].

Finally, patients with WD require strict follow-up, frequently including visits to various specialists due to the heterogeneous nature of the disease [16], which can impact their health-related quality of life (HRQoL) and impose a high economic burden on the National Health System (NHS) [17]. Nevertheless, data documenting the impact of the disease on patient’s HRQoL, productivity, or healthcare resource utilisation (HRU) are scarce.

In view of the above, we aimed to assess the HRQoL of patients with WD in a real-world setting in Spain, evaluating the impact of the disease on their lives. In addition, we aimed to determine treatment adherence, drug preference and satisfaction, HRU, and productivity losses.

## 2. Materials and Methods

### 2.1. Study Design

We carried out a multicentric, cross-sectional, retrospective, observational study in four tertiary Spanish hospitals.

Variables were collected at the inclusion visit by investigators and patients, as well as retrospectively from medical records by the investigators. All data were recorded after patients had signed a specific informed consent.

### 2.2. Patients

Adults aged ≥ 18 years old and adolescents of 12 years or older diagnosed with WD were included in the study if there were data available in their electronic medical history from at least one year before inclusion and if they understood and were able to answer the study questionnaires properly, according to medical opinion. WD diagnosis was confirmed if the Leipzig score was ≥4 points [18].

Patients currently presenting with WD-related acute liver failure or those who had received a liver transplantation were not considered.

### 2.3. Research Variables and Instruments

Sociodemographic (age, gender, employment status) and clinical variables (weight, height, family history of WD, genotype, current phenotype and WD phenotype at diagnosis [18], current laboratory and elastography data, time since WD diagnosis, first and current treatment, time on current treatment, concomitant comorbidities and therapies) were collected from medical records. Maintenance therapy was considered if it was received more than 12 months apart from the diagnosis.

Additional information on the questionnaires and the ad hoc questionnaire used in the study are provided in the supplementary material.

The Unified Wilson’s Disease Rating Scale (UWDRS) was used by physicians with experience in the use of the scale and in research to score functional and neurological impairments at the inclusion visit as described elsewhere [19]. The higher the UWDRS score, the higher the neurological impairment.

Patients’ HRQoL was measured using the validated Spanish version of the EuroQoL-5D-5L (EQ-5D-5L) questionnaire, which evaluates the generic quality of life in five dimensions [20], as well as self-reported overall health status rated on a visual analogue scale (EQ-VAS). A higher EQ-VAS score identified patients with a better HRQoL.

The impact of WD on a patient’s life was measured using ad hoc questionnaires based on the 5-point Likert scale questions adapted from Dress et al. [21], including questions on the difficulty experienced in performing activities of daily living, the impact of the disease on social life, emotional status, and executive function.

Patients’ treatment satisfaction and preferences were evaluated using an ad hoc questionnaire that included Likert scale or dichotomic questions to assess their degree of agreement with different statements related to their WD treatment.

Treatment adherence was assessed through the Simplified Medication Adherence Questionnaire (SMAQ) [22] and using an ad hoc questionnaire based on Likert scale and dichotomic questions to assess self-reported adherence. The SMAQ consists of six questions that evaluate different aspects of patient compliance with treatment. A patient is classified as “non-adherent” if they respond to any of the questions with a non-adherence answer or if the patient had missed more than two doses during the last week or not taken medication on more than two whole days during the last three months.

Data were collected on patients’ HRU (visits to the specialist, visits to other professionals paid for by the patient, hospitalizations, and visits to the emergency department). Their work/academic productivity during the previous year and their associated costs were calculated. Work productivity was assessed in employed patients using the validated Spanish version of the Work Productivity and Activity Impairment (WPAI) questionnaire [23]. It consists of six questions related to work productivity and impairment of general activity, expressed as a percentage. Similarly, academic performance was assessed in school patients using the Classroom Impairment Questionnaire (CIQ) [24], comprising three questions about classroom productivity.

The supplementary material includes additional information about the questionnaires and the ad hoc questionnaires used in the study.

### 2.4. Sample Size Calculation

Sample size was estimated based on the mean estimation of the EQ-5D-5L VAS score [25], the Spanish population size in 2020 (47,332,614) [26], and disease prevalence (3.3/100,000 inhabitants) [2,3,4,27]. Based on these data, the target population comprised 1562 patients (N). Assuming the conservative standard deviation of 24.25 that was previously published, a confidence level of 95%, a precision error of 5%, and the finite population correction [28], the sample size required to accomplish the main objective of this study was 86 patients.

### 2.5. Data Analysis

A descriptive analysis of the study variables was performed. Relative and absolute frequencies were calculated to describe qualitative variables. Centrality and dispersion measures (mean, standard deviation [SD], quartiles, minimum, and maximum) were calculated for quantitative variables.

To analyse the impact of WD on a patient’s daily life, the answers to the ad hoc questionnaire were classified as (1) none and small difficulty, (2) some difficulty, and (3) difficult and extremely difficult, or as (1) never and rarely, (2) occasionally, and (3) most of the time and all time. Similarly, for patient self-reported treatment satisfaction and preferences, answers were classified as (1) agree and strongly agree and (2) disagree and strongly disagree.

The analysis was performed for the whole patient population and separately by subgroups according to their predominant condition at the study visit. Group H (for “hepatic”) included patients with isolated liver involvement; group EH (for “extra-hepatic”) included patients with neurological or mixed (neurological and liver) involvement. The differences between both subgroups were statistically incomparable as the sample size in each subgroup was not large enough. Thus, the analysis was only descriptive.

Cost analysis included direct costs (visits to NHS specialists, emergency departments, and hospitalizations), indirect costs (loss of productivity), and out-of-pocket costs (specialists paid for by patients). The costs of nursing homes and medications were not included.

Data analysis was performed using the software STATA v.14.

## 3. Results

### 3.1. Patient Characteristics

A total of 102 patients were included in the study (85.3% adults and 14.7% adolescents). The participants’ mean (SD) age was 36.06 (15.1) years old and 59 (57.8%) of them were male. The mean (SD) time from diagnosis was 20.50 (11.85) years; 40 (39.2%) patients reported a family history of WD.

Group H included 83 (81.4%) patients while group EH included 19 (18.6%) individuals, 15 (14.7%) with mixed involvement and four (3.9%) with the isolated neurological phenotype. See Table 1 for more details.

The mean (SD) of the total UWDRS questionnaire score at the inclusion visit was 8.19 (18.06). Functional and neurological impairment was higher in group EH [33.68 (30.38)] than in group H [2.35 (3.73)]. Overall, the liver severity was mild. Cirrhosis was present in only 15 (14.7%) patients; most of them (n = 14, 93.3%) were classified as Child-Pugh Class A, and the mean Model for End-Stage Liver Disease score was 7.2. Five of the cirrhotic patients currently had oesophageal varices while none of the patients had a current or past history of hepatic decompensation.

### 3.2. HRQoL

Patients reported a mean (SD) EQ-VAS score of 80.75 (17.43) points, which was higher for group H [83.81 (SD: 14.03)] than for group EH [67.37 (SD: 24.0)]. Anxiety or depression (42.2%) and pain or discomfort (32.4%) were the dimensions of HRQoL most frequently affected.

The impact of the disease on HRQoL domains was higher in patients from group EH. Nevertheless, more than 40% of the patients in group H reported being at least slightly anxious or depressed due to WD (Table 2).

### 3.3. Impact on Patients’ Lives

#### 3.3.1. Activities in Daily Life

Most patients (86.3–95.1%) evaluated the impact of WD on daily activities as *not at all* or *some difficulty*. The daily activities most often evaluated as *extremely difficult* or *very difficult* were “going to work” and “doing physical activity or exercise”.

**Figure 1:**
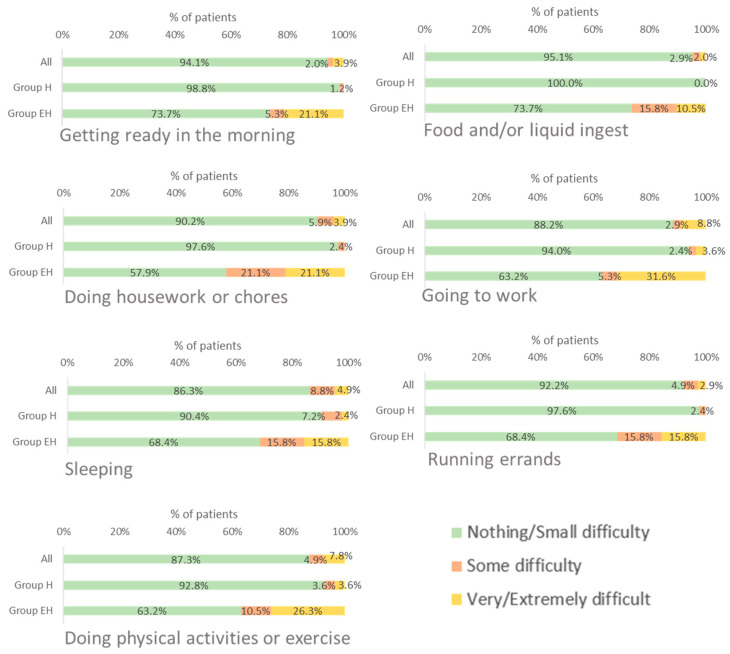
Difficulties experienced by WD patients with daily life activities. EH: mixed or neurological involvement; H: isolated liver involvement.

A higher percentage of patients in group EH reported greater difficulty in carrying out all daily activities compared with patients in group H (Figure 1).

#### 3.3.2. Emotional Impact

**Figure 2:**
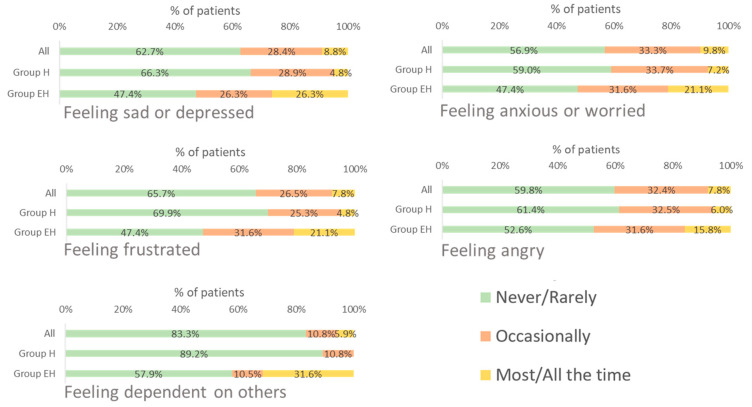
Frequency of impact of WD on the emotional life domain. EH: mixed or neurological involvement; H: isolated liver involvement.

Approximately 30% of the patients considered that the disease *occasionally* affected all their emotional dimensions. The emotional dimensions most frequently impacted were feeling anxious or worried and sad or depressed (Figure 2).

A higher proportion of patients in group EH, compared with patients in group H, reported that the disease impacted all dimensions of the emotional domain *most*/*all the time*, with over 50% of patients feeling sad or depressed, anxious or worried, and frustrated *occasionally* or *most/all the time* (Figure 2).

#### 3.3.3. Social Impact

**Figure 3:**
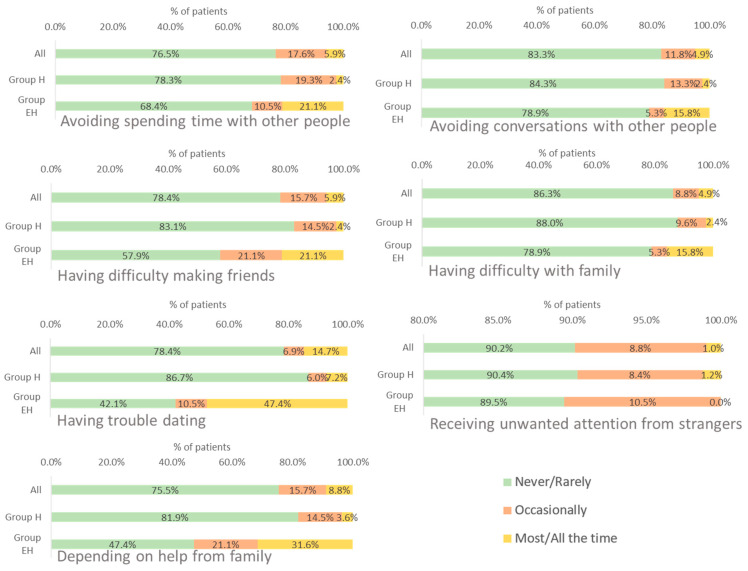
Frequency of impact of WD on patients’ social activities. EH: mixed or neurological involvement; H: isolated liver involvement.

Most of the patients (75.5–90.2%) reported that the disease *never* or *rarely* impacted their social life. The social dimensions most frequently evaluated as *most/all the time* were “having trouble in dating” and “depending on help from family”. For both dimensions this percentage was higher in patients in group EH (47.7% and 31.6%, respectively) than those in group H (7.2% and 3.6%, respectively) (Figure 3).

#### 3.3.4. Executive Function

Over 80% of the patients reported that the disease *never* or *rarely* affected their executive function. The area most affected *most*/*all the time* was “having difficulty coping with multiple tasks”.

**Figure 4:**
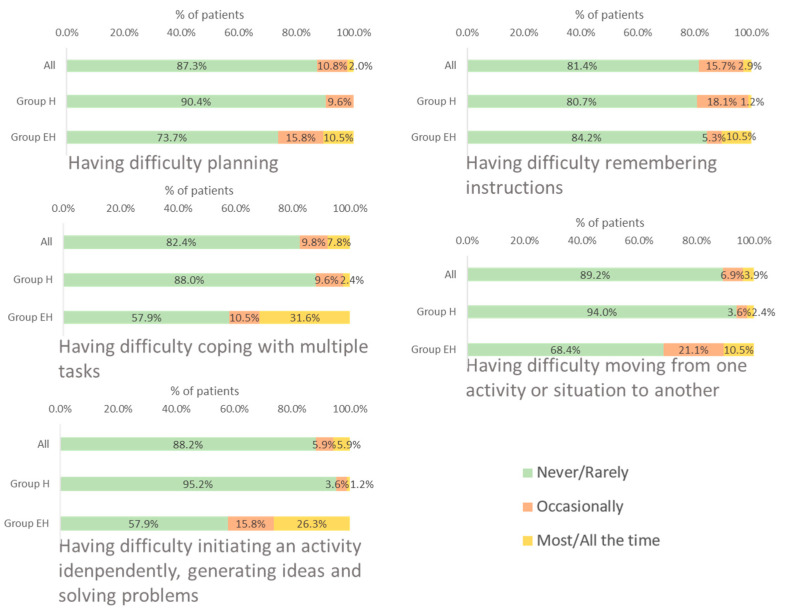
Frequency of impact of WD on patients’ executive function. EH: mixed or neurological involvement; H: isolated liver involvement.

The executive function was more affected in group EH patients than in group H, especially when initiating an activity independently, generating ideas and solving problems, and coping with multiple tasks. However, a slightly higher proportion of patients in group H reported having difficulty remembering instructions *occasionally* or *most/all the time* than those in group EH (19.3% vs. 15.8%) (Figure 4).

### 3.4. Treatment Evaluation

Zinc salts were the most frequent current treatment in the overall patient population (48.0%) and in both subgroups (group H: 45.8%; EH: 57.9%). The mean (SD) time with the current treatment at the inclusion visit was 9.58 (9.7) years. Most patients (88.2%, n = 90) were in the maintenance phase of therapy; approximately half of the patients (56.4%, n = 57) had previously received a different treatment for WD, and the most frequent reason for treatment change was “following the recommendation of the clinical protocol” (66.7%) (Table 3).

The most common first treatment in patients with WD overall and by subgroups was D-penicillamine (57.8%), followed by zinc salts (31.4%) (Table S1).

A total of 47 (46.1%) patients of the cohort received concomitant treatments. The most frequent treatment in the cohort was anti-hypertensive therapy (12.7%), followed by anxiolytics (11.8%), antidepressants (8.8%), and other neuropsychiatric drugs (such as hypnotics and sedatives, antiepileptics, antipsychotics, antiparkinsonians, and narcotic and psychotropic drugs) (12.8%). In this regard, 12 (14.5%) patients in group H and 10 (52.6%) in group EH were treated with at least one of the aforementioned neuropsychiatric drugs. Details of the concomitant treatments prescribed in patients with WD are shown in Figure S1.

#### 3.4.1. Treatment Satisfaction and Preferences

Over 70% of patients were *satisfied* or *totally satisfied* with their current treatment. A total of 33 (32.4%) patients experienced side effects. Of these, approximately 60% reported that side effects *somewhat* or *considerably* affected their satisfaction with the treatment (Table 4).

Most patients (57.9%) reported that their treatment required taking too many pills daily. In this respect, 88.7% of patients *agreed* or *strongly agreed* on the preference to take fewer pills during the day if possible while 63.8% of them would agree to change their current treatment if they could (Figure S2).

The subgroups analysis showed that a higher agreement was reached in group H than in group EH regarding the difficulty of complying with the medication regimen (40.5% vs. 15.8%) and the need to take too many pills daily (61.8% vs. 42.1%) (Figure S2).

#### 3.4.2. Treatment Adherence

According to the SMAQ, only 23 (22.5%) patients of the cohort were fully adherent to their therapy: 15 (18.1%) in group H and 8 (42.1%) in group EH.

Regarding self-reported adherence, 70 (68.7%) patients [54 (65.1%) in group H, 16 (84.2%) in group EH] considered that their current treatment was *easy* or *very easy* to adhere to. However, a total of 69 (67.6%) patients reported frequently forgetting pills, with this fact being more frequent in patients in group H (72.3%, n = 60) than in group EH (47.4%, n = 9) (Table S2).

### 3.5. HRU and Associated Costs

#### 3.5.1. Ambulatory and Specialist Visits

The mean (SD) number of ambulatory visits during the previous year was 4.7 (3.5) and was slightly higher in patients in group EH (5.6, SD: 2.5) compared with those in group H (4.4, SD: 3.6).

Due to the study design and recruitment responsibilities, most of the patients were visited by hepatologists (96.1%, n = 98), followed by neurologists (43.1%, n = 44) and ophthalmologists (26.5%, n = 27) (Table 5).

The mean (SD) number of visits not included in the NHS was 34.4 (51.3) and was much higher among patients in group EH (74.3, SD: 61.7) compared with those in group H (7.8, SD: 14.2), with an especially high number of physiotherapy and speech therapy visits (Table 5).

#### 3.5.2. Emergency Visits and Hospitalization

Emergency services were required once by eight (7.8%) patients and twice by four (3.9%) patients; only one visit was related to WD. Hospitalization was needed once by four (3.9%) patients and twice by two (2.0%) patients but was unrelated to WD in half of the hospitalised patients. The overall mean (SD) of the hospital length of stay was 2.7 (1.2) days.

Up to seven (6.9%) patients (six of whom belong to the EH group) needed a caregiver. Five (4.9%) patients (one in group H and four in group EH) required an informal caregiver; two (2.0%) patients from group EH received care supported by the NHS and two (2.0%) stayed in a nursing home.

#### 3.5.3. Productivity

Employees and students missed 3.6% of work and 1.5% of school time, respectively, due to the disease (absenteeism). In addition, employees and students reported that the disease affected their productivity (presenteeism) by 6.7% and 5.4%, respectively. The percentage of overall work impairment due to the disease (absenteeism and presenteeism) was 7.8% for employees and 6.9% for students, being higher in employed patients in group EH (20.4%) than in group H (6.1%) (Table S3).

#### 3.5.4. Cost Analysis

Finally, the median of total cost per month was EUR 70.60 and was higher in group EH than in group H (EUR 114.90 vs. EUR 59.62).

## 4. Discussion

Our study evaluated the HRQoL of patients with WD and how the disease impacts different dimensions of their lives, and determined patients’ treatment adherence, preference, and satisfaction. In addition, we assessed the HRU and economic burden of the disease in the Spanish context.

To the best of our knowledge, this is the first real-world study carried out in a Spanish WD population evaluating both the humanistic and economic impacts of the disease.

Patients’ characteristics included in this study (58% male; highly enriched with isolated hepatic phenotypes, 81.4%) might be explained by the origin of the study design, which was developed by hepatologists, and are similar to those described in other studies in Europe [29,30,31,32]. It is important to mention that most of the patients included in the study had non-advanced liver disease, and most of the neurological/mixed phenotypes had a low UWDRS score at the inclusion visit. Therefore, overall, this WD cohort was enriched with patients with mild disease.

Nevertheless, 42.2% and 32.4% of all patients reported anxiety or depression and pain or discomfort, respectively, based on the results of the EQ-5D-5L questionnaire. Overall, a greater impact on HRQoL was found among patients in group EH than those in group H; however, patients in both subgroups reported a similar impact in the anxiety or depression domain.

Data on the HRQoL of patients with WD in the literature are scarce. In line with our results, previous studies have shown lower HRQoL scores in patients with WD and neurological and psychiatric symptoms compared with patients presenting only hepatic involvement [31,33,34,35,36]. Compared with a European study with pooled normative EQ-5D-3L data (mean EQ-VAS: 78.3) [37], our entire cohort showed a similar EQ-VAS value (80.75). A previous study with a Spanish population showed an EQ-VAS value slightly lower than ours (73.4). However, the mean age of this population was higher than ours, which could explain this difference [38]. A recently published study of 257 patients with WD reported an EQ-VAS value of 75.1, slightly lower than ours. This difference could be explained by the highest proportion of patients with the hepatoneurologic phenotype included in this study (39.3%) [36].

In this context, the patients in our study, particularly those in group EH, reported that the disease mostly impacted their emotional status, followed by their social life (for example, in dating). Thus, at least one in three patients in each subgroup reported feeling depressed, anxious, frustrated, or angry daily. These results are similar to those found in a previous study in which patients with WD reported that the disease impacted both their social life and their ability to work or their academic performance, and also restricted their physical activity [39]. Furthermore, the study reported that patients stated they were embarrassed and worried about how others perceived them [39], which could lead to anxiety or frustration. Our results also confirm those obtained by Dress et al., who reported that emotional status was the domain most negatively affected by the disease among patients with WD [21].

As expected, most of our cohort’s patients were initially treated with D-penicillamine at diagnosis. At the inclusion visit for this study, however, zinc salts were predominant, mostly due to a common local protocol switch to maintenance therapies. Most patients in our study reported being satisfied with their treatment. Notably, 77.5% of the patients were found to be non-compliant with therapy when assessed with the SMAQ, even though just 35.7% of the patients had indicated that the medication regimen was difficult to comply with. This finding reinforces the need for more patient-friendly drug formulations. Our compliance results are similar to those observed in a recent French cross-sectional study in which only 21% of patients showed high treatment adherence, assessed using the Morisky scale [40]. In contrast, studies in the Polish population showed 72–74% treatment adherence [41,42]. These differences could be explained, at least partially, by the different adherence measurements used in different studies, or by the type and severity of the disease. The subgroup analysis revealed that patients in group H were less adherent (18.1% vs. 42.1%) and had greater difficulty in complying (40.5% vs. 15.8%) than those in group EH. This might be explained by a reduced symptomatic phenotype with less consciousness of the disease in group H compared with group EH patients. Clinicians should be aware of this and promote treatment adherence, especially in patients with milder forms of the disease, as treatment discontinuation may lead to symptoms worsening or even death [41,43].

It is worth noting that neuropsychiatric drugs were commonly prescribed as concomitant treatments (21.6%) in our population, with more frequent use among patients in group EH (52.6%) than those in group H (14.5%). Overall, 8.8% required antidepressants, which is higher than the prevalence of antidepressant intake reported for the Spanish population (5.5%) [44]. The use of antidepressants in group H was lower (3.6%) than in the EH group (31.6%); however, a high incidence of other neuropsychiatric drugs, such as anxiolytics and hypnotics, was observed in patients with isolated hepatic involvement. Considering all together, this information should raise awareness of the emotional impact of the disease and the consequent use of neuropsychiatric drugs, together with the high need for multidisciplinary follow-up.

Finally, regarding HRU for patients with WD, data in the literature are scarce. A case-control study conducted in the US showed an increase in HRU and healthcare costs in patients with WD compared with the period before being diagnosed and compared with matched chronic liver disease controls [45]. Similarly, our study found that the mean number of visits within and outside the NHS was high, especially among patients in group EH compared with those in group H. Consequently, the health cost per month was higher for patients in group EH. Overall, WD imposes a high resource and economic burden on both patients and the NHS. Therefore, new treatments with fewer adverse events could help to reduce the economic burden of the disease. However, the great challenge is to establish early diagnosis to achieve lower copper toxicity and milder presentations of the disease.

Our study presents some strengths. A total of 102 patients were included in the study, constituting a large cohort considering WD prevalence. All variables were analysed in the entire cohort and in subgroups according to their clinical phenotype. Furthermore, the study included both physicians’ and patients’ perspectives, thus obtaining a more complete vision of the disease. However, the study also presents some limitations. Patients were recruited in four hospitals around Spain, mainly from three big cities on the mainland (Barcelona, Madrid, Valencia), and differences between subgroups were only numerical, as sample size did not allow statistical comparisons; therefore, extrapolation of these results to the rest of the country and the whole WD population should be made with caution. The questionnaire used was based on an ad hoc, unvalidated questionnaire that was previously published. We also lacked a control group of healthy individuals of the same age and sex, thus preventing comparison with the global Spanish population. Finally, we cannot omit the possibility of the post-pandemic economic crisis having an influence on our results.

## 5. Conclusions

In conclusion, here we present a comprehensive study of 102 patients with WD in a real-world setting in Spain. Patients with WD showed a deteriorated HRQoL, with high rates of depression and anxiety. This emotional impact is aggravated in patients with hepatic and neurological involvement. Most patients are satisfied with their treatment; however, they reported taking too many pills and would change their treatment if possible. Low treatment adherence is reported, highlighting the need for other therapies favouring adherence.

Moreover, patients with WD require a high HRU within and outside the NHS, imposing a high economic burden on our society.

Our study provides a complete overview of the WD situation in Spain and contributes to improving disease awareness and guidance for the management of patients with WD.

## Figures and Tables

**Table 1:** Sociodemographic and clinical characteristics of all patients in the study and by subgroups (n = 102).

Variable	All	Group H (n = 83)	Group EH (n = 19)
**Age in years, mean (SD)**	36.06 (15.14)	34.90 (14.97)	41.1 (15.25)
**Weight in kg, mean (SD)**	69.12 (13.61)	70.44 (13.27)	63.37 (13.94)
**BMI**			
Underweight, n (%)	9 (8.8)	5 (6.0)	4 (21.1)
Normal, n (%)	56 (54.9)	46 (55.4)	10 (52.6)
Overweight, n (%)	32 (31.4)	28 (33.7)	4 (21.1)
Obese, n (%)	5 (4.9)	4 (4.8)	1 (5.3)
**Gender**			
Male, n (%)	59 (57.8)	51 (61.4)	8 (42.1)
Female, n (%)	43 (42.2)	32 (38.6)	11 (57.9)
**Employment status**			
Student, n (%)	24 (23.5)	23 (27.7)	1 (5.3)
Employed, n (%)	49 (48.0)	43 (51.8)	6 (31.6)
Unemployed *, n (%)	12 (11.8)	11 (13.3)	1 (5.3)
Disabled, n (%)	12 (11.8)	3 (3.6)	9 (47.4)
Retired, n (%)	3 (2.9)	1 (1.2)	2 (10.5)
Temporarily disabled, n (%)	2 (2.0)	2 (2.4)	0 (0.0)
**Laboratory test**			
Alanine amino transferase (U/L), mean (SD)	49.9 (48.7)	53.4 (52.6)	34.6 (18.5)
Aspartate amino transferase (U/L), mean (SD)	36.8 (23.3)	38.4 (24.9)	29.9 (12.8)
Platelets (10^9^/L), mean (SD)	242.1 (85.3)	249.4 (85.5)	210.3 (78.9)
**Time since diagnosis in years, mean (SD)**	20.5 (11.8)	19.98 (11.5)	22.75 (13.3)
**Phenotype at WD diagnosis** [18]			
Acute hepatic presentation, n (%)	13 (12.7)	10 (12.0)	3 (15.8)
Chronic hepatic presentation, n (%)	67 (65.7)	63 (75.9)	4 (21.1)
Neurological presentation associated with symptomatic liver disease, n (%)	9 (8.8)	1 (1.2)	8 (42.1)
Neurological presentation not associated with symptomatic liver disease, n (%)	7 (6.96)	3 (3.6)	4 (21.1)
Neurological presentation with the presence or absence of liver disease not investigated, n (%)	1 (1.0)	1 (1.2)	0 (0.0)
Other presentations, n (%)	5 (4.9)	5 (6.0)	0 (0.0)
**UWDRS**	8.2 (18.1)	2.3 (3.7)	33.7 (30.4)
**Total, N (%)**	102 (100)	83 (100)	19 (100)

BMI: body mass index; EH: mixed or neurological involvement; H: isolated liver involvement; SD: standard deviation; UWDRS: Unified Wilson’s Disease Rating Scale. * The unemployed category indicates patients who are unemployed and/or seeking employment.

**Table 2:** Distribution of reported problems in each dimension of the EQ-5D-5L questionnaire.

Dimension	Statement	All	Group H	Group EH
		N (%)	N (%)	N (%)
**Mobility**	I have no problems in walking about	83 (81.4)	76 (91.6)	7 (36.8)
	I have slight problems in walking about	10 (9.8)	4 (4.8)	6 (31.6)
	I have moderate problems in walking about	7 (6.9)	3 (3.6)	4 (21.1)
	I have severe problems in walking about	1 (1.0)	0 (0.0)	1 (5.3)
	I am unable to walk about	1 (1.0)	0 (0.0)	1 (5.3)
**Self-care**	I have no problems washing or dressing myself	91 (89.2)	79 (95.2)	12 (63.2)
	I have slight problems with washing or dressing myself	7 (6.9)	4 (4.8)	3 (15.8)
	I have moderate problems washing or dressing myself	2 (2.0)	0 (0.0)	2 (10.5)
	I have severe problems washing or dressing myself	0 (0.0)	0 (0.0)	0 (0.0)
	I am unable to wash or dress myself	2 (2.0)	0 (0.0)	2 (10.5)
**Usual activities**	I have no problems in doing my usual activities	79 (77.5)	72 (86.7)	7 (36.8)
	I have mild problems in doing my usual activities	12 (11.8)	9 (10.8)	3 (15.8)
	I have moderate problems in doing my usual activities	7 (6.9)	2 (2.4)	5 (26.3)
	I have severe problems in doing my usual activities	2 (2.0)	0 (0.0)	2 (10.5)
	I am unable to do my usual activities	2 (2.0)	0 (0.0)	2 (10.5)
**Pain or discomfort**	I have no pain or discomfort	69 (67.6)	61 (73.5)	8 (42.1)
	I have slight pain or discomfort	22 (21.6)	15 (18.1)	7 (36.8)
	I have moderate pain or discomfort	10 (9.8)	6 (7.2)	4 (21.1)
	I have severe pain or discomfort	1 (1.0)	1 (1.2)	0 (0.0)
	I have extreme pain or discomfort	0 (0.0)	0 (0.0)	0 (0.0)
**Anxiety or depression**	I am not anxious or depressed	59 (57.8)	48 (57.8)	11 (57.9)
	I am slightly anxious or depressed	29 (28.4)	25 (30.1)	4 (21.1)
	I am moderately anxious or depressed	10 (9.8)	9 (10.8)	1 (5.3)
	I am very anxious or depressed	4 (3.9)	1 (1.2)	3 (15.8)
	I am extremely anxious or depressed	0 (0.0)	0 (0.0)	0 (0.0)
**Total**		102 (100)	83 (100.0)	19 (100.0)

EH: mixed or neurological involvement; H: isolated liver involvement.

**Table 3:** Characteristics of treatment in all patients with WD and by subgroups.

Variable	All	Group H	Group EH
**Current WD treatment (chelators/non chelators)**			
D-penicillamine, n (%)	33 (32.4)	28 (33.7)	5 (26.3)
D-penicillamine + Zinc, n (%)	6 (5.9)	6 (7.2)	0 (0.0)
Trientine, n (%)	7 (6.9)	5 (6.0)	2 (10.5)
Trientine + Zinc, n (%)	3 (2.9)	2 (2.4)	1 (5.3)
Zinc, n (%)	49 (48.0)	38 (45.8)	11 (57.9)
Others *, n (%)	4 (3.9)	4 (4.8)	0 (0.0)
**Time with current treatment in years, mean (SD)**	9.58 (9.77)	8.89 (8.62)	12.76 (13.81)
**Maintenance phase (>12 months since Dg), n (%)**	90 (88.2)	73 (88.0)	17 (89.5)
Different previous treatment, n ** (%)	57 (56.4)	48 (57.8)	9 (50.0)
**Reasons for the drug change**			
Clinical protocol, n (%)	38 (66.7)	31 (64.6)	7 (77.8)
Adverse event, n (%)	11 (19.3)	9 (18.8)	2 (22.2)
Therapeutic failure, n (%)	6 (10.5)	6 (12.5)	0 (0.0)
Other reasons, n (%)	2 (3.5)	2 (4.2)	0 (0.0)
**Current concomitant treatments (yes), n (%)**	47 (46.1)	35 (42.2)	12 (63.2)

EH: mixed or neurological involvement; H: isolated liver involvement; SD: standard deviation. * bis-choline tetrathiomolybdate; ** Previous treatment corresponds to patients who have received any treatment for WD other than the current one.

**Table 4:** Distribution of the responses to the treatment satisfaction questionnaire by all patients and by subgroups.

Question	Treatment Satisfaction				
**How much is your overall satisfaction with your current WD treatment?**	**Totally unsatisfied**	**Unsatisfied**	**Indifferent**	**Satisfied**	**Totally satisfied**
All, n (%)	5 (4.9)	9 (8.8)	12 (11.8)	39 (38.2)	37 (36.3)
Group H, n (%)	4 (4.8)	7 (8.4)	12 (14.5)	28 (33.7)	32 (38.6)
Group EH, n (%)	1 (5.3)	2 (10.5)	0 (0.0)	11 (57.9)	5 (26.3)
**To what extent do these side effects affect your satisfaction with the treatment?**	**Totally**	**Considerable**	**Somewhat**	**Minimally**	**Not at all**
All, n (%)	0 (0.0)	6 (18.2)	13 (39.4)	11 (33.3)	3 (9.1)
Group H, n (%)	0 (0.0)	5 (17.9)	11 (39.3)	10 (35.7)	2 (7.1)
Group EH, n (%)	0 (0.0)	1 (20.0)	2 (40.0)	1 (20.0)	1 (20.0)

EH: mixed or neurological involvement; H: isolated liver involvement; n: number of patients.

**Table 5:** Mean number of visits to each medical specialty within and outside the National Health System.

	All		Group H		Group EH	
	Mean (SD)	n	Mean (SD)	n	Mean (SD)	n
**Visits within the National Health System**						
Hepatology/Digestive	3.3 (2.6)	98	3.2 (2.8)	79	3.7 (2.0)	19
Psychiatry	2.3 (2.0)	6	2.8 (2.4)	4	1.5 (0.7)	2
Neurology	1.7 (0.9)	44	1.7 (0.9)	27	1.6 (0.8)	17
Ophthalmology	1.4 (1.0)	27	1.5 (1.2)	21	1.0 (0.0)	6
Number of visits to specialists (not including others)	4.5 (3.4)	100	4.2 (3.6)	81	5.6 (2.5)	19
Number of visits to other specialists	2.1 (1.6)	8	2.1 (1.6)	8	-	-
Total number of visits to specialists	4.7 (3.5)	100	4.4 (3.6)	81	5.6 (2.5)	19
**Visits outside the National Health System**						
Physiotherapy	42.5 (29.1)	8	13.5 (14.9)	2	52.2 (26.4)	6
Speech therapy	63.5 (22.3)	4	-	-	63.5 (22.3)	4
Optometry	1.7 (1.6)	6	3.0 (2.8)	2	1.0 (0.0)	4
Odontology	1.8 (1.4)	8	1.0 (0.0)	5	3.0 (1.7)	3
Number of visits to specialists (not including others)	38.6 (55.3)	16	4.8 (7.9)	8	72.5 (62.2)	8
Number of visits to other specialists	11.7 (18.0)	6	14.0 (22.8)	4	7.0 (1.4)	2
Total number of visits to specialists	34.4 (51.3)	20	7.8 (14.2)	12	74.3 (61.7)	8

EH: mixed or neurological involvement; H: isolated liver involvement; SD: standard deviation.

## Data Availability

Qualified academic investigators may request participant-level, de-identified clinical data and supporting documents (statistical analysis plan and protocol) pertaining to this study. Further details regarding data availability, instructions for requesting information and our data disclosure policy will be available on the Alexion.com website (http://alexion.com/responsibility, accessed on 27 June 2023).

## Supplementary Materials

The following supporting information can be downloaded at: https://www.mdpi.com/article/10.3390/jcm12144823/s1, Figure S1: Frequency of concomitant treatment in patients with WD. * The group of “other medicines” was related to hormone drugs or vitamin supplements. One patient could be treated with more than one concomitant treatment; Figure S2: Frequency of the degree of agreement with the items in the preference questionnaire; Table S1: Frequency of first treatment in all patients and by subgroup; Table S2: Evaluation of physician-determined and self-reported treatment adherence; Table S3: Productivity evaluation according to the Work Productivity and Activity Impairment questionnaire.
