# Supplementary material for: Health-Related Quality of Life in Patients Living with Wilson Disease in Spain: A Cross-Sectional Observational Study
Source: J Clin Med. 2023 Jul 21;12(14):4823. doi: 10.3390/jcm12144823 (PMC10381913; doi:10.3390/jcm12144823)
Supplement: Supplementary file 1 [file jcm-12-04823-s001.zip › jcm-2502961-supplementary.pdf]

Supplementary Materials

Health-related quality of life in patients living with Wilson disease in Spain: a cross-sectional observational study

Zoe Mariño[1]*, Marina Berenguer[2], Luis Peña-Quintana[3,4], Antonio Olveira[5], Anna Miralpeix[1], Isabel Sastre[2], Ana Reyes-Domínguez[3], Pilar Castillo[5], Clàudia García-Solà[1], Ariadna Bono[2], Miriam Romero[5], Francisco Javier Pérez-Sádaba[6], Susana Aceituno[6], Anna Anguera[7]

1 Liver Unit, Hospital Clínic Barcelona, IDIBAPS, CIBERehd, ERN-RARE Liver, University of Barcelona, Barcelona, Spain; ZMARINO@clinic.cat (Z.M.); AMIRALPEIX@clinic.cat (A.M.); CLGARCIAS@clinic.cat (C.G-S.)
2 Department of Gastroenterology and Hepatology, Hospital Universitari i politècnic La Fe, CIBERehd and IISLaFe, Valencia, Spain; marina.berenguer@uv.es (M.B.); isa.sastre99@gmail.com (I.S.); ariadna_bono@iislafe.es (A.B.)
3 Pediatric Gastroenterology, Hepatoloy and Nutrition Unit, Complejo Hospitalario Universitario Insular Materno-Infantil, Las Palmas de Gran Canaria, Spain; luis.pena@ulpgc.es (L.P-Q.); anitard71@gmail.com (A.R-D.)
4 ACIP. CIBEROBN, Instituto de Salud Carlos III, University of Las Palmas de Gran Canaria, Spain; luis.pena@ulpgc.es (L.P-Q.)
5 Department of Gastroenterology, Hospital La Paz, Madrid, Spain; aolveiram@gmail.com (A.O.); pcastillograu@gmail.com (P.C.); miriam.romero.portales@gmail.com (M.R.)
6 Outcomes'10 SLU, Castellón de la Plana, Spain; fperez@outcomes10.com (F.J.P-S.); saceituno@outcomes10.com (S.A.)
7 Medical Department, Alexion, AstraZeneca Rare Diseases, Spain; Anna.Anguera@alexion.com (A.A.)
* Correspondence: ZMARINO@clinic.cat

Research variables and instruments

- Unified Wilson's Disease Rating Scale consists of three subscales representing three main features of clinical manifestation in neurological WD: UWDRS I (1 item), UWDRS II (14 items), UWDRS III (19 items). Each item is scored on a five-point scale (0 points "no symptoms" and 4 points "worst characteristic possible") except for the first item, in which the maximum score is 3 points.
- The EuroQoL-5D-5L evaluates five dimensions: mobility, self-care, usual activities, pain/discomfort, and anxiety/depression. Each dimension has three levels of severity, corresponding to: 1) no problems, 2) slight problems, 3) moderate problems, 4) severe problems, and 5) extreme

problems. EQ-VAS ranging from 0 “worst possible” to 10 “best possible” health.

- The *ad hoc* questionnaire to evaluate the impact of WD on the patient’s life included questions ranging from 1) “not at all difficult” to 5) “extremely difficult” for questions about activities of daily living and from 1) “never” to 5) “all the time” for the rest of questions.
- SMAQ consists of six questions that evaluate different aspects of patient compliance with treatment: forgetfulness, routine, adverse effects, and quantification of omissions. A patient is classified as non-adherent if they respond to any of the questions with a non-adherence answer or if the patient has lost more than two doses during the last week or has not taken medication during more than two complete days during the last three months.
- WPAI consists of six questions related to the numbers of hours missed from work and usual activities, providing scores expressed as percentages for absenteeism, presenteeism, overall work impairment, and impairment of general activity. Using a scale from 0) “no effect” to 10) “health issues completely prevented me from doing work”.
- The CIQ comprises three questions about hours missed from class because of health issues, class hours attended, and how much health issues affected classroom productivity while attending classes. Using a scale from 0) “no effect” to 10) “health issues completely prevented me from doing work”.

<u>Ad-hoc questionnaire</u>

WD impact on patient's life (ADL, social, emotional, and executive function)

Activities of daily living impacts

	Not at all difficult	A little bit difficult	Somewhat difficult	Very diffi-cult	Extremely difficult
Getting ready in the morning	☐	☐	☐	☐	☐
Swallowing foods and/or liquids	☐	☐	☐	☐	☐
Running errands	☐	☐	☐	☐	☐
Doing housework or chores	☐	☐	☐	☐	☐
Going to work	☐	☐	☐	☐	☐
Physical activity or exercise	☐	☐	☐	☐	☐
Sleeping	☐	☐	☐	☐	☐

Emotional impacts

	Never	Rarely	Occasion-ally	Most of the time	All of the time
Feeling sad or de-pressed	☐	☐	☐	☐	☐
Feeling anxious or worried	☐	☐	☐	☐	☐
Feeling frustrated	☐	☐	☐	☐	☐
Feeling angry	☐	☐	☐	☐	☐
Feeling dependent on others	☐	☐	☐	☐	☐

Social impacts

	Never	Rarely	Occasion-ally	Most of the time	All of the time
Avoid spending time with other people	☐	☐	☐	☐	☐
Avoid conversa-tions with others	☐	☐	☐	☐	☐
Difficulty making friends	☐	☐	☐	☐	☐
Difficulty with my family	☐	☐	☐	☐	☐
Trouble dating	☐	☐	☐	☐	☐
Receiving un-wanted attention from strangers	☐	☐	☐	☐	☐
Relying on family for help	☐	☐	☐	☐	☐

Executive function impacts

	Never	Rarely	Occasion-ally	Most of the time	All of the time
**Difficulty planning**	☐	☐	☐	☐	☐
**Difficulty remem-bering instructions**	☐	☐	☐	☐	☐
**Difficulty juggling multiple tasks**	☐	☐	☐	☐	☐
**Difficulty moving from one activity or situation to an-other**	☐	☐	☐	☐	☐
**Difficulty inde-pendently begin-ning an activity, generating ideas, and solving prob-lems**	☐	☐	☐	☐	☐

Satisfaction, adherence & preferences regarding treatment *ad hoc* questionnaire (satisfaction questions adapted from TSQM)

SATISFACTION WITH WD TREATMENT:

- How satisfied are you overall with your current WD treatment?

Totally unsatisfied	Unsatisfied	Indifferent	Satisfied	Totally satisfied

As a result of taking medication, do you experience any side effects? ☐YES ☐NO

- If ☒***YES*** , rate your satisfaction with the treatment side effects experienced during treatment?

Totally unsatisfied	Unsatisfied	Indifferent	Satisfied	Totally satisfied

ADHERENCE TO WD TREATMENT:

- How easy or difficult is it to follow the treatment you receive currently?

Very difficult	Difficult	Somewhat easy	Easy	Very easy

- Do you ever forget to take your medication? ☐YES ☐NO
- Do you ever decide not to take your medication? ☐YES ☐NO
- During the last week, how many times did you fail to take your medication as prescribed? ☐Never ☐1-2 times ☐more than twice

PREFERENCES REGARDING WD TREATMENT:

Please indicate how strongly you agree with the following statements:

- I believe the treatment I receive is safe: Strongly agree/agree/disagree/strongly disagree/NA.
- I consider it necessary to monitor the copper levels through labs and another tests: Strongly agree/agree/disagree/strongly disagree/NA.
- Controlling my copper levels is a major burden on my daily life: Strongly agree/agree/disagree/strongly disagree/NA.
- I know how to take my medication correctly: Strongly agree/agree/disagree/strongly disagree/NA.

- My medication regimen is difficult to comply with: Strongly agree/agree/disagree/strongly disagree/NA.
- I needed to stop treatment due to side effects: Strongly agree/agree/disagree/strongly disagree/NA.
- I take too may pills daily: Strongly agree/agree/disagree/strongly disagree/NA.
- If I could, I would take fewer pills daily: Strongly agree/agree/disagree/strongly disagree/NA.

Figure S1. Frequency of concomitant treatment in patients with WD. *The group of “other medicines” was related to hormone drugs or vitamin supplements. One patient could be treated with more than one concomitant treatment.

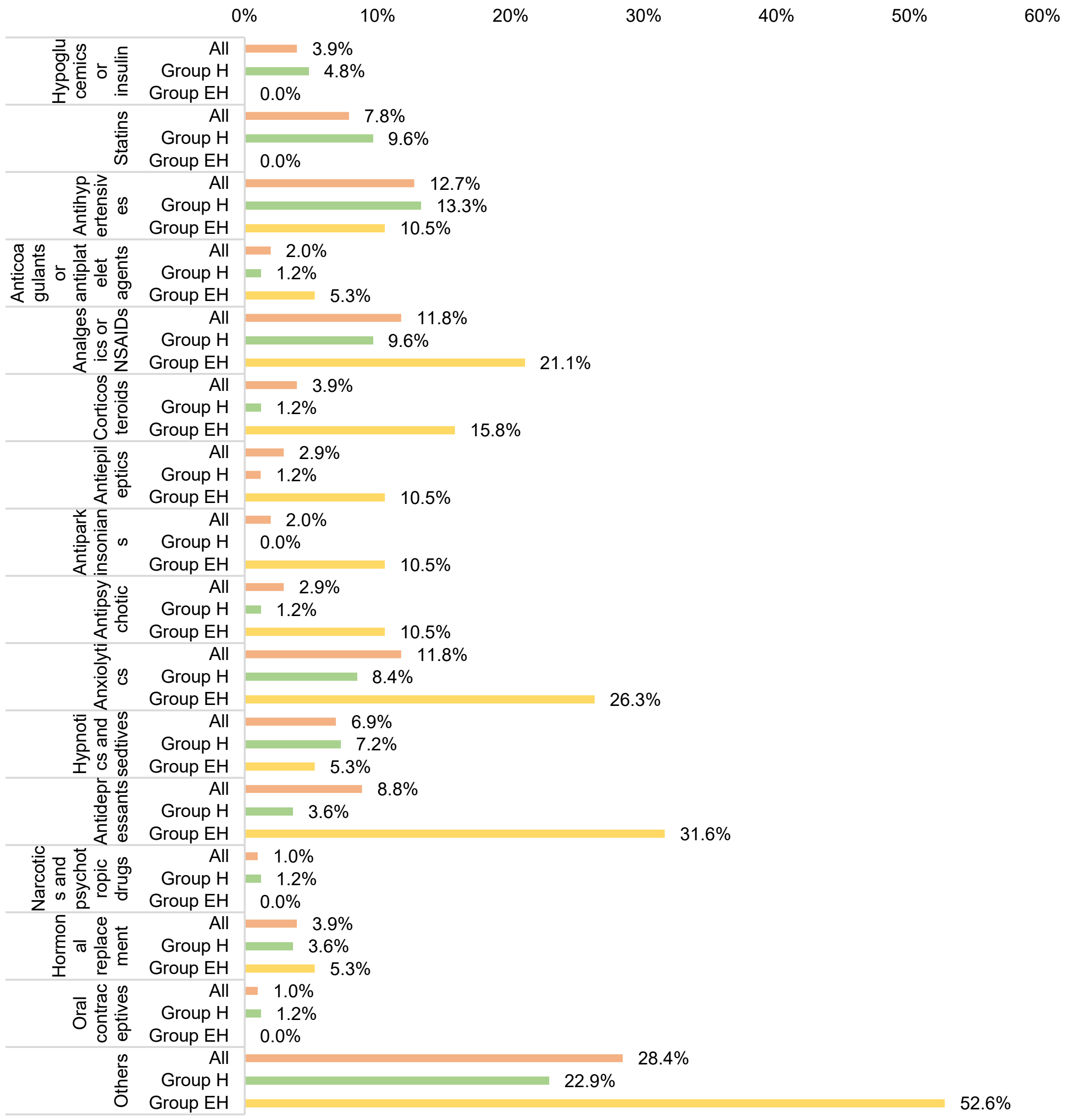

Figure S2. Frequency of the degree of agreement with the items in the preference questionnaire

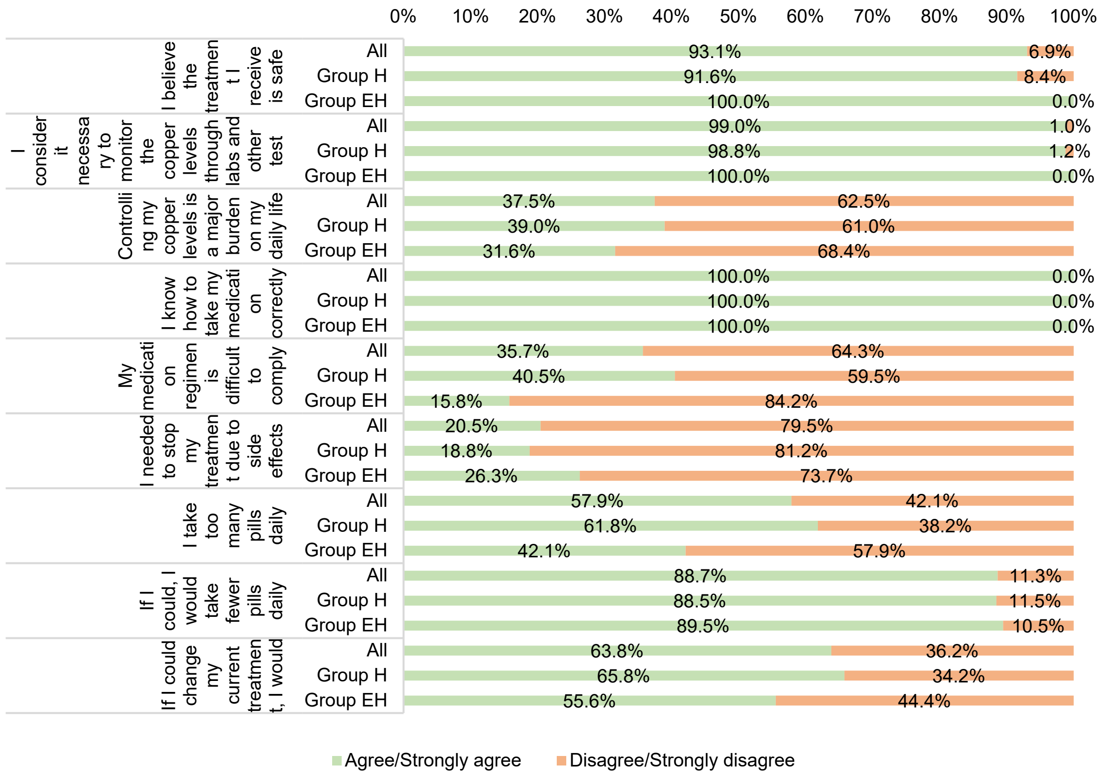

Table S1. Frequency of first treatment in all patients and by subgroup.

First WD treatment	All	Group H	Group EH
**D-penicillamine, n (%)**	59 (57.8)	46 (55.4)	13 (68.4)
**D-penicillamine + Zinc, n (%)**	7 (6.9)	5 (6.0)	2 (10.5)
**Trientine, n (%)**	1 (1.0)	0 (0.0)	1 (5.3)
**Trientine + Zinc, n (%)**	2 (2.0)	1 (1.2)	1 (5.3)
**Zinc, n (%)**	32 (31.4)	30 (36.1)	2 (10.5)
**Others*, n (%)**	1 (1.0)	1 (1.2)	0 (0.0)
**Total, N (%)**	102 (100.0)	83 (100.0)	19 (100.0)

EH: mixed or neurological involvement; H: isolated liver involvement. * Bis-choline tetrathiomolybdate.

Table S2. Evaluation of physician-determined and self-reported treatment adherence

	All		Group H		Group EH	
	**Yes**	**No**	**Yes**	**No**	**Yes**	**No**
	**N (%)**	**N (%)**	**N (%)**	**N (%)**	**N (%)**	**N (%)**
**SMAQ**						
Have you ever forgotten to take your medication?	70 (68.6)	32 (31.4)	61 (73.5)	22 (26.5)	9 (47.4)	10 (52.6)
Do you always take your medication at the appropriate time?	55 (53.9)	47 (46.1)	41 (49.4)	42 (50.6)	14 (73.7)	5 (26.3)
When you feel bad, have you ever discontinued taking your medication?	13 (12.7)	89 (87.3)	11 (13.3)	72 (86.7)	2 (10.5)	17 (89.5)
Have you ever forgotten to take your medication during the weekend?	20 (19.6)	82 (80.4)	19 (22.9)	64 (77.1)	1 (5.3)	18 (94.7)
**Self-reported**						
Have you ever forgotten to take your medication?	69 (67.6)	33 (32.4)	60 (72.3)	23 (27.7)	9 (47.4)	10 (52.6)
Do you forget to take your medication over the weekend?	25 (24.5)	77 (75.5)	21 (25.3)	62 (74.7)	4 (21.1)	15 (78.9)
Do you ever voluntarily decide not to take your medication?	12 (11.8)	90 (88.2)	8 (9.6)	75 (90.4)	4 (21.1)	15 (78.9)
Did you always take your medication during the last week?	61 (59.8)	41 (40.2)	47 (56.6)	36 (43.4)	14 (73.7)	5 (26.3)
Did you fail to take your medication once or twice during the last week?	34 (33.3)	68 (66.7)	31 (37.3)	52 (62.7)	3 (15.8)	16 (84.2)
Did you fail to take your medication more than twice during the last week?	7 (6.9)	95 (93.1)	5 (6.0)	78 (94.0)	2 (10.5)	17 (89.5)

EH: mixed or neurological involvement; H: isolated liver involvement; SMAQ: Simplified Medication Adherence Questionnaire

Table S3. Productivity evaluation according to the Work Productivity and Activity Impairment questionnaire

	All		Group H		Group EH	
	**Mean % (SD)**	**n**	**Mean % (SD)**	**n**	**Mean % (SD)**	**n**
**Percentage of work time missed due to the disease**	3.6 (0.2)	51	3.1 (15.5)	45	6.9 (10.8)	6
**Percentage of how your illness affected your productivity while you were working**	6.7 (0.2)	51	5.3 (17.0)	45	16.7 (27.3)	6
**Percentage of overall work impairment due to the disease**	7.8 (0.2)	51	6.1 (17.9)	45	20.4 (30.8)	6
**Percentage of school time missed due to the disease**	1.5 (0.04)	26	1.6 (4.4)	24	0.0 (0.0)	2
**Percentage of how your illness affected your productivity while being at school due to the disease**	5.4 (0.2)	26	1.3 (3.4)	24	55.0 (63.6)	2
**Percentage of overall school impairment due to the disease**	6.9 (0.2)	26	2.9 (5.2)	24	55.0 (63.6)	2
**Percentage of regular daily activity impairment due to the disease**	14.7 (0.3)	102	9.0 (18.5)	83	39.5 (40.6)	19

EH: mixed or neurological involvement; H: isolated liver involvement; SD: standard deviation
